# An enhanced partial order curve comparison algorithm and its application to analyzing protein folding trajectories

**DOI:** 10.1186/1471-2105-9-344

**Published:** 2008-08-18

**Authors:** Hong Sun, Hakan Ferhatosmanoglu, Motonori Ota, Yusu Wang

**Affiliations:** 1 Department of Computer Science and Engineering, The Ohio State University, Columbus, OH 43210, USA; 2 Global Scientific Information and Computing Center Tokyo Institute of Technology, O-okayama, Meguro-ku, Tokyo 152-8550, Japan

## Abstract

**Background:**

Understanding how proteins fold is essential to our quest in discovering how life works at the molecular level. Current computation power enables researchers to produce a huge amount of folding simulation data. Hence there is a pressing need to be able to interpret and identify novel folding features from them.

**Results:**

In this paper, we model each folding trajectory as a multi-dimensional curve. We then develop an effective multiple curve comparison (MCC) algorithm, called the *enhanced partial order (EPO) *algorithm, to extract features from a set of diverse folding trajectories, including both successful and unsuccessful simulation runs. The EPO algorithm addresses several new challenges presented by comparing high dimensional curves coming from folding trajectories. A detailed case study on miniprotein Trp-cage [1] demonstrates that our algorithm can detect similarities at rather low level, and extract biologically meaningful folding events.

**Conclusion:**

The EPO algorithm is general and applicable to a wide range of applications. We demonstrate its generality and effectiveness by applying it to aligning multiple protein structures with low similarities. For user's convenience, we provide a web server for the algorithm at .

## Background

Proteins are the main agents in cells. From a chemical point of view, a protein molecule is a linear sequence of amino acids. This linear sequence, under appropriate physicochemical conditions, folds into a unique native structure rapidly. Understanding folding process is of paramount importance, especially since the outcome of it, namely the three dimensional protein structure, to a large extent decides the functionality of the molecule. Hence a lot of research has been devoted to investigating the kinetics of protein folding. In particular, modern (parallel) computation power makes it possible to perform large-scale folding simulations. As a result, interpreting the huge amount of simulation data obtained becomes a crucial issue.

Given the highly stochastic nature of the protein motion, the study of protein fold usually relies on an ensemble of folding simulations including both *successful *and *unsuccessful *runs, which are trajectories that do or do not include a sequence of conformations leading to a near native conformation. Given such a diverse data set, scientists wish to answer questions such as what causes the folding process falling into different results, and what common properties are shared by the successful runs, but not the unsuccessful ones? To this end, it is highly desirable to be able to compare multiple folding trajectories and extract useful information from them.

In this paper, we model each protein folding trajectory as a multi-dimensional curve, and then present a novel multiple-curve comparison (MCC) algorithm to identify critical information from a set of trajectory curves in an automatic manner. In particular, we focus on the geometry of protein chain conformations throughout the folding process, and convert each conformation into a high dimensional point. The goal is to extract lists of *ordered events *common to successful runs but not to unsuccessful ones, such as discovering that a conformation *B *is always formed after *A *and followed by a conformation *C *before reaching a successful folding conformation. (Conformations *A*, *B*, and *C *may not be consecutive.) To this end, we develop an effective new multiple curves comparison algorithm called the *enhanced partial order (EPO) *algorithm, to capture similarities and dis-similarities between a set of input folding trajectories. The EPO algorithm is developed over the concept of POA (partial order alignment) [2,3], but is greatly improved and extended in several aspects, especially in its sensitivity in detecting low level of similarity and its capability of handling high dimensional curves. Applying it to the folding trajectories of a miniprotein Trp-cage [1] shows that our algorithm is able to automatically detect important critical folding events which are observed earlier [4] by biological methods. Our EPO algorithm is general, and we demonstrate its generality and effectiveness by also applying it to aligning multiple protein structures with low similarities.

### Related work

Previously, folding simulations analysis is performed mainly for testing various protein folding models [5-7], such as the folding pathway model and the funnel model; and/or for studying energetic aspects of folding kinetics [8-11]. The geometric shapes of the conformations involved in folding trajectories have not been widely explored [4,12,13], despite their important role in folding. A particularly interesting work in this direction is by Ota et al. [4], where they investigated the folding trajectories of a mini-protein Trp-cage using phylogenic tree combined with expert knowledge. However in general, an automatic tool to facilitate the folding simulations analysis at large scales is still missing. This paper provides an important step towards this goal by modeling folding trajectories as curves and using a new multiple curve comparison (MCC) algorithm to detect critical folding events.

The closest relative of our MCC problem in computational biology is the multiple structure alignment (MSTA) problem, which aims at aligning a family of protein structures, each modeled as a three dimensional polygonal curve to represent its backbone.

MSTA is a very hard problem. In fact, even the pairwise comparison problem of aligning two structures *A *and *B *is believed to be NP-hard since one has to optimize simultaneously both the correspondence between *A *and *B *and the relative transformation of one structure with respect to the other. Numerous heuristic-based algorithms have been developed in practice for this fundamental problem [14-20]. If we have a set of *k *> 0 structures, then even the problem of aligning them optimally *without *considering transformations becomes intractable – it takes Ω(*n*^*k*^) time using the standard dynamic programming algorithm, where *n *is the size of each protein involved.

In practice, progressive methods are widely used to attack the MSTA problem [21]. For example, given a set of structures, many approaches start with a seed structure and then progressively align the remaining structures onto it one by one [22-28]. A consensus or core structure is typically built throughout, to maintain the common substructures among the proteins that are already aligned. At each round, usually only pairwise structure comparison is performed to align the current consensus with a new structure.

The above progressive MSTA framework is a greedy approach. Its performance depends on the underlying pairwise comparison methods used, the order of structures that are progressively aligned, as well as the consensus structure maintained. Various heuristics have been exploited to find a good order for the progressive alignments. Note that this order can also be guided by a tree instead of a linear sequence, which removes the need of choosing a seed structure. The progressive procedure may also be iterated several times to locally refine the multiple structure alignments.

### Our results

There are two main differences between the MCC problem we are interested in and the traditional MSTA problem. In the case of protein structures, it is usually explicitly or implicitly assumed that the (majority of the) input proteins belong to one family (How to classify a set of input structures into different families is a related problem, and many such classifications exist [17,29,30]), or at least share some relations. As such, one can expect that some consensus of the family should exist. However in our case, the set of curves are from a set of simulations including both successful and unsuccessful runs, and we wish to classify this *diverse *set of curves, and capture common features within as well as across its sub-families. Secondly and more importantly, the level of similarity existing in these folding trajectories is usually much lower than that in a family of related proteins. Hence we aim at an algorithm with high sensitivity, which is able to detect small-scaled partial similarity and handle multi-dimensional curves (trajectories) as well.

**Figure 1:**
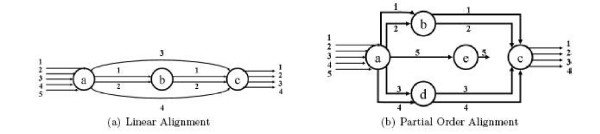
**Linear graph vs. partial order graph**. Aligning five trajectories (IDs 1 to 5) using (a) a linear graph, and (b) a partial order graph. Symbols in the circles are the node IDs and numbers on edges are trajectory IDs. Note that the linear alignment in (a) will not be able to record the partial similarity between curves 3 and 4, which is maintained in (b) (i.e, node *d*).

In this paper, we propose and develop a sensitive MCC algorithm, called the *EPO (enhanced partial order) *algorithm, to compare a set of diverse high dimensional curves. Our algorithm follows a similar framework as the POA algorithm [3,28] to encode the similarities of aligned curves in a partial order graph, instead of in a linear structure used by many traditional MSTA algorithms. This has the advantage that other than similarities among all curves, similarities among a subset of input curves can also be encoded in this graph. See Figure 1 for an example, where nodes in both graphs represent a group of aligned points from input curves.

For the more important problem of sensitivity, we observe that being a greedy approach, the progressive MSTA framework tends to be inherently insensitive to low level of similarities – if one early local decision is wrong, it may completely miss a small-scaled partial similarity. To improve this aspect of the performance of the progressive framework, we first propose a novel two-level scoring function to measure similarity, which, together with a clustering idea, greatly enhances the quality of the local pairwise alignment produced at each round. We then develop an effective merging step to post-process the obtained alignments. This step helps to reassemble vertices (high dimensional points) from input curves that should be matched together, but were scattered in several sub-clusters in the alignments due to some earlier non-optimal decisions. Both techniques are general and can be used to improve the performance of many existing MSTA algorithms. Experimental results show that our MCC algorithm is highly sensitive and able to classify input curves. We also demonstrate the power of our tool in mining critical events from protein folding trajectories using a detailed case study of a miniprotein Trp-cage.

Although our EPO algorithm is developed with the goal of comparing folding trajectories, the algorithm is general and can be applied to other domains as well, such as protein structures or pedestrian trajectories extracted from surveillance videos [31]. In particular, in this paper, we demonstrate this generality by showing that the EPO algorithm can be used to improve the results of existing multiple protein structure alignment algorithms, especially when input proteins share low structural similarity.

## Results

### Experimental Results on trajectory data

In this section, we report a systematic performance study on a biological dataset that contains 200 molecular dynamics simulations. The experiments achieve the following goals: First, we show that the quality of the alignments produced by our EPO algorithm is significantly better than that of the original POA algorithm. Second, we demonstrate the effectiveness of our algorithm by applying it to real protein simulation data and obtaining biologically meaningful results that are consistent with previous discoveries [4].

#### Background of dataset

**Figure 2:**
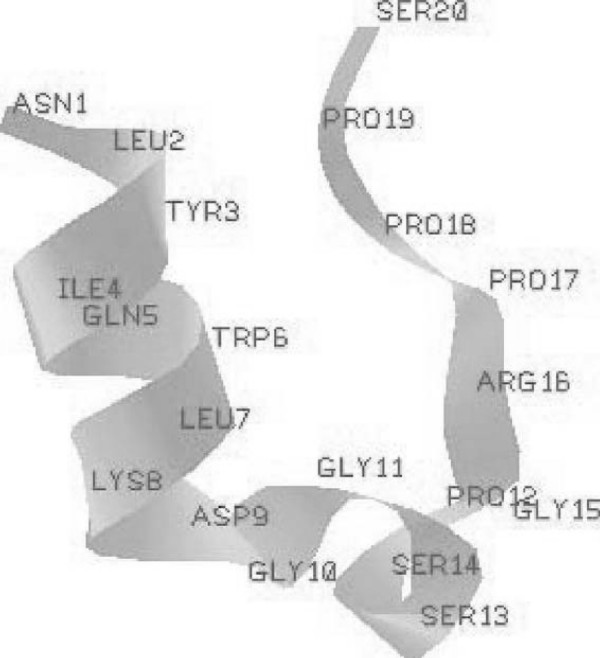
**NMR structure of trp-cage protein **1l2y. Labels on graph mark amino acids(AAs). AA2 to AA7 roughly form an alpha-helix. AA2 to AA19 form a ring-type structure. In particular, AA2 to AA5 and AA16 to AA19 form the "neck" of this ring.

Our input dataset includes 200 simulated folding trajectories for a particular protein called Trp-cage. The dataset is provided by the Ota's Lab [4]. The folding simulations were performed at 325 K by using the AMBER99 force field with a small modification and the generalized Born implicit solvent model. Trp-cage (see Figure 2) is a mini-protein consisting of 20 amino acids. It has been widely used for folding study because of its short, simple sequence and its quick folding kinetics. Following the definition from [32], a successful folding event has to satisfy the following two criteria:

• The RMSD for a conformation from the native NMR structure [1] is less than 2 Å.

• A subsequence of such near-native conformations holds for at least 200 ps.

In [4], 58 successful folding trajectories reaching successful folding events are identified, and each trajectory includes 101 successive conformations sampled at 20 ps interval. Furthermore, there are two crucial observations in [4] that we will examine in the our experiment. First, before moving to the native conformation, a "ring" sub-structure (see Figure 2) has to be formed. Second, the distinction of native and pseudonative confirmations heavily relies on side-chain position of "ring" sub-structure. Ota et al. [4] obtained the above results by aligning each pair of trajectories first and then applying a neighbor joining method to group similar trajectories together. However this semi-automatic approach requires dedicated expert knowledge. The following experiments applied on the same dataset show that our EPO algorithm can automatically detect the above folding events with little prior knowledge.

#### Experimental setting

In order to be consistent with the results from [4], we select all 58 successful folding events, and call it *SuccData*. We also randomly select 58 unsuccessful folding trajectories, each containing 101 conformations, and collect them in a set called *FailData*. The union of successful and unsuccessful data is referred to as the *MixData*. There are two parameters used in our experiments: *ε*, and *τ*. They are set as *ε *= 1 *Å *and *τ *= 40 in all our experiments unless specified otherwise. Their meaning will be explained in Section 3 Method.

#### Investigation on entire protein structure

**Figure 3:**
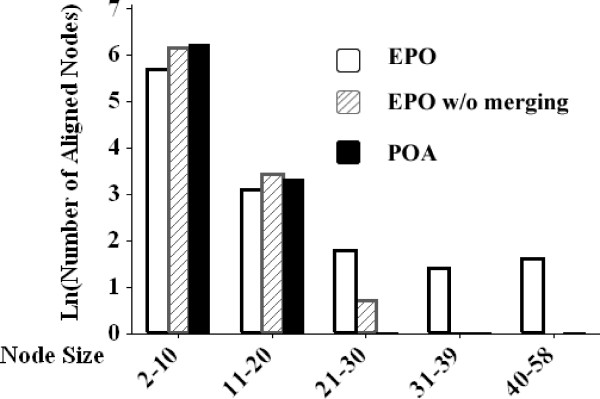
**Comparison of EPO, EPO-NoMerge and POA**. Distribution of aligned nodes produced by the EPO algorithm, EPO-NoMerge (i.e, first stage of the EPO algorithm), and the traditional POA algorithm. The histogram is the number of aligned nodes (*y*-axis) versus the size of aligned nodes (*x*-axis).

In the first set of experiments, we convert each conformation to a high dimensional point (i.e, a 20 × 20 = 400 dimensional point), based on the distance matrix between all of the alpha-carbon atoms. Figure 3 compares the quality of the alignments of the *SuccData *by performing the POA algorithm, our EPO algorithm without the merging procedure (EPO-NoMerge), and the EPO algorithm. It shows the number of aligned nodes (y-axis) versus the size of aligned nodes (x-axis). Note that EPO-NoMerge is essentially POA with a clustering preprocessing and the new two-level scoring function.

The similarity level between these trajectories is low (i.e, the number of aligned nodes with large size is small). It is clear from this histogram that our EPO algorithm significantly outperforms the other two by producing more aligned nodes with large sizes. The comparison between EPO and EPO-NoMerge demonstrates the effectiveness of our merging procedure, and that EPO-NoMerge is better than POA shows that the *two-level scoring function *as well as the *clustering preprocessing *greatly enhances the performance. We have also performed experiments which show that compared to the POA algorithm, EPO-NoMerge is much less sensitive to the order of curves aligned. Comparisons of the three algorithms over the *MixData *produces a similar result, and majority of points aligned to heavy nodes (i.e, |*o*| ≥ 40) are from successful runs (the results are not shown in this report).

We also observe that most of the heavily aligned nodes are close to the end of the trajectories for the *SuccData*. In fact, many aligned points have conformation IDs around and greater than 90, which is indeed the time that the folding starts to get stabilized. More specifically, consider the set of aligned nodes of size greater than 40 for the *SuccData*. Among all points aligned to these nodes, 67.2% has a conformation ID greater than 90, and 24.4% has an ID between 80 and 90. This implies that our algorithm has the potential to detect the stabilization of successful folding events in an automatic manner.

This also implies that using the entire protein structure may be too coarse to detect critical folding events, as they are usually induced by small key motifs. In what follows, we map only a substructure of the input protein into a multi-dimensional point and provide more detailed analysis of this folding data.

#### Investigation on substructures

It is usually believed that certain critical motifs play important roles which stabilize the whole structure in the folding process [6,7]. We wish to have a tool that can identify such critical motifs (substructures) automatically. We define a candidate motif to be two subchains of Trp-cage, each of length 4. These two pieces induce a sub-window in the distance map of each conformation of the protein. We further require that the number of contacts in this subwindow w.r.t. the distance map of the native structure is at least 4, where a contact corresponds to two alpha-carbon atoms within distance 6 *Å*. We collect a set of candidate motifs based on these criteria.

Now for a candidate motif, for each of its conformation of along the trajectory, we consider the distance matrix between its alpha-carbon atoms as before, and convert the folding trajectory of this motif into a curve in the 4 × 4 = 16 dimensional space. In order to be more discriminative, we also introduce a *side-chain weighting factor α*, ranging from 0 to 1, to include the side chain information when comparing two high dimensional points: *α *= 0 means that side-chain information is completely ignored (Roughly speaking, for every conformation, we record for each residue also the relative position of the centroid of its side-chain with respect to its alpha-carbon atom. This provides another high dimensional point that we call a *side-chain point*. The distance between two conformations will combine the distance between their side-chain points by the side-chain weighting factor). We perform our EPO algorithm on both the *SuccData *and the *MixData*, and there are two motifs that especially stand out, which we describe below.

#### Alpha-helix substructure

The first one corresponds to an alpha-helix substructure. In Figure 2, five successive amino acids (No.2–7) form an alpha-helix structure which is a simple, self-contained secondary structure (SSE) [1]. From the results returned by our EPO algorithm, we note that this alpha-helix is formed rather early consistently in both successful and unsuccessful runs. Once formed, it remains stable. This is consistent with the common conception that due to its chemical property, alpha-helix is a stable secondary structure, and can be formed quickly. Hence the formation of alpha-helix cannot be used to differentiate successful runs from unsuccessful ones.

#### Ring-substructure

The second motif corresponds to the neck of a ring structure. In particular, it consists of the sub-chains of No. 2 – 5 and No. 16 – 19 amino acids. The following results demonstrate that EPO can automatically not only find but also track the formation of such fingerprint sub-structures (critical motif).

First, we observe from Table 1 that when applying the EPO algorithm to the *MixData *(with the sidechain weight factor *α *= 0.9), signifificant alignments involve mainly trajectories from *SuccData*. For example, the last row of Table 1 shows that among the 62 points (from 62 trajectories) aligned to a particular node, 58 are from *SuccData*, with the remaining 4 from *FailData*. Hence this motif is potentially critical to the success of the folding of Trp-cage. It also suggests that we can automatically classify the *MixData *into *SuccData *and *FailData *with few false positives based on this ring-neck motif, while previously, the classification in the input data was obtained by a few expert defined rules.

**Table 1:** EPO on ring structure(MixData).

		Classification		
			
Aligned Node ID	|*o*_*i*_| ≥ *τ*	*SuccData*	*FailData*	D(o) Å
1	49	27	22	1.852
2	45	28	17	1.798
3	41	29	12	2.189
4	40	31	9	1.447
5	48	31	16	1.761
6	40	32	8	1.322
7	47	34	13	1.133
8	42	35	7	1.923
9	44	36	8	0.873
10	49	42	7	1.428
11	54	48	6	1.020
12	59	50	9	1.294
13	60	51	9	0.932
14	56	52	4	1.255
15	62	56	6	1.782
16	62	58	4	1.503

Column 2 – 4 shows the size of an aligned node (i.e, the number of points aligned to this node) from *MixData, SuccData*, and *FailData*, respectively. Column 4 shows the diameter of this node (note that the distance threshold *ε *= 1 *Å *means that the diameter of a node can be up to 2 *Å*).

Second, when the side-chain weighting factor *α *= 0.9, it turns out that 49.6% of significant aligned nodes are formed before the conformation ID 85 (compared to results from Section). For example, there are two aligned nodes from the successful runs, where 80% of points (i.e. trajectories) aligned to them has a conformation ID between 75 – 85. This implies that the complete formation of this ring-neck usually immediately precedes the stabilization of the folding structure (which is roughly at conformation ID 90 for successful trajectories).

If reducing the side-chain weighting factor *α *to 0.5, naturally, we found more aligned nodes. In particular, other than the cluster with conformations of IDs around 80, we observe more significant clusters involving conformations with IDs from 50–70. By comparing the conformations of the ring-neck motif in these clusters with those in the aligned nodes around 80, we found that the backbone structures are rather similar, but the side-chains are of different orientations. In other words, the shape of the ring-neck motif is first stabilized by the backbone structure, and then the side-chains gradually move into right position. There are a few trajectories where the side-chains eventually move to the mirror image of their correct positions, and lead to pseudo-native conformations which can be detected when considering the side-chains.

**Figure 4:**
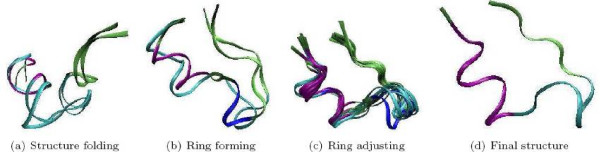
**Folding events**. Visualizing of vital events listed the Table 1 during the folding procedure. Purple: *α*-helix, blue: 3 – 10-helix, cyan: turn, lime: coil. Corresponding to the Table 1, the alignment node IDs in: (a)-(1, 2), (b)-(3, 4), (c)-(5–15), (d)-(16).

Figure 4 displays several groups of critical events identified by the EPO algorithm (corresponding to the aligned nodes as shown in Table 1). In particular, 4(a) includes two closely occurred events during the early stage of the folding procedure (one of the conformations is selected from the aligned node 1, and the other one is from aligned-node 2). At this time, the sequence has started to fold and one can observe the helical structure, but the ring is not yet formed. 4(b) presents two conformations representing aligned nodes 3 and 4, respectively. We observe that the ring has started to form at this point, but is still not stable. 4(c) shows several conformations (one each from aligned nodes 5 to 15) occurred in order in most successful runs. At this stage, the ring is adjusted and stabilized. The adjustment mainly happens around the turn area and for side chains. 4(d) shows the final successful structure.

The above results are consistent with the results from [4], where such a ring-shaped substructure was discovered semi-automatically by pairwise structure comparisons together with expert knowledge.

#### Timing of EPO

The above experiments are implemented on a Windows XP machine with 1.5 GHz CPU and 512 MB Memory. Table 2 compared the running time of the three methods: EPO, EPO without merging, and the POA algorithm (re-implemented by us). The EPO algorithm is faster than the traditional POA algorithm. This is because EPO pre-processes all points from input curves by clustering them into groups and only creates a new aligned node if it has a good potential to be heavily aligned. Thus it is not surprising that the EPO algorithm takes less time in aligning *Faildata *than *SuccData*, while the POA algorithm takes longer time (as it creates a lot of aligned nodes, leading to large partial order graphs). This interesting property implies that the EPO algorithm is effective at aligning curves with low level of similarities.

**Table 2:** Comparing processing-time.

	*MixData*	*SuccData*	*Faildata*
	unit = Min.		

EPO	≈ 30	≈ 14	≈ 7
EPO w/o merging	≈ 26	≈ 12	≈ 6
POA	≈ 49	≈ 23	≈ 33

Comparing processing-time by the EPO algorithm, EPO-NoMerge and the traditional POA algorithm in three datsets. *MixData *includes 116 trajectories, *SuccData *includes 58 trajectories and *FailData *includes 58 trajectories.

### Experimental Results on protein structure data

Although the EPO algorithm was primarily developed for multiple high dimensional trajectories alignment, it can be used to improve multiple protein structures alignment as well. In particular, assume that we are given an alignment of a set of protein structures, that is, a transformation for each protein involved, as well as the correspondences between their residues. We can apply our EPO algorithm to further improve the correspondences based on the given transformations. We now use the EPO algorithm to refine the correspondences returned by four current popular multiple structure alignment algorithms: CE-ME [23], Multiprot [33], MAMMOTH [34] and POSA [28]. Table 3 shows our testing data set, which are the common sets used in previous multiple protein structure comparison algorithms. Set 1 contains several Calcium-binding proteins that have rather similar structures. Set 2 and set 3 contain a more diverse set of calmodulin-like proteins and tim-barrels proteins, respectively.

**Table 3:** Protein structural data set. "Average Size" is the average number of residues in each protein.

Data Set ID	Number of Structures	Average Size	PDB Codes
Set 1			
Calcium-binding	6	140	4cpv2scpA2sas1top1scmB3icb
Set 2			
Calmodulin-like	3	161	1jfjA1ncx2sas
Set 3			
Tim-barrels	7	391	1btc1pii1tml4enl5rubA6xia7timA

Table 4 shows the results of applying EPO to the alignments of Calcium-binding proteins (set 1) as returned by other MSTA algorithms. "NCORE" refers to the number of residues aligned from each protein and "RMSD" refers to average root-mean square deviation of the aligned core. Note that as the six proteins in the data set have very similar structures, most algorithms can already align them rather well. Nevertheless, EPO still marginally improve the quantity and quality of alignment except for a slight increase in the RMSD when applied to the alignment returned by MAMMOTH. We test the EPO results by different ordering of input proteins. The results are consistent, which suggests that our EPO algorithm is robust.

**Table 4:** Results of refinement on calcium-binding proteins(Set 1) across 4 algorithms.

	Before EPO refinement		After EPO refinement	
	
	NCORE	RMSD	NCORE	RMSD
CE-MC	57	3.02 Å	63	2.80 Å
Multiprot	48	1.66 Å	52	1.56 Å
MAMMOTH	14	1.01 Å	15	1.09 Å
POSA	56	2.82 Å	59	2.78 Å

Table 5 and 6 show the results of applying the EPO refinement to two structurally-diverse sets of proteins whose structural cores are harder to detect. There are notable improvements in terms of both quantity and quality.

**Table 5:** Results of refinement on calmodulin-like proteins(set 2) across 4 algorithms.

	Before EPO refinement		After EPO refinement	
	
	NCORE	RMSD	NCORE	RMSD
CE-MC	62	5.85 Å	77	4.77 Å
Multiprot	56	1.93 Å	60	1.90 Å
MAMMOTH	15	0.94 Å	17	0.85 Å
POSA	65	2.67 Å	68	2.34 Å

**Table 6:** Results of refinement on tim-barrels proteins(Set 3) across 2 algorithms(CE-MC and MAMMOTH failed to return any aligned core).

	Before EPO refinement		After EPO refinement	
	
	NCORE	RMSD(Å)	NCORE	RMSD(Å)
Multiprot	24	1.82	29	1.83
POSA	40	3.6	50	3.22

In summary, the above results are similar to the case of comparing multiple folding trajectories. In particular, the two-stage scoring function exploited by our EPO algorithm is effective and alleviates the ordering issue in progressive MSTA. The merging stage helps to produce better correspondences between input structures and makes the algorithm robust. Hence the EPO algorithm can be used as a post-processing tool for current MSTA softwares to refine and optimize their alignment results.

## Discussion and conclusion

In this paper we proposed and developed EPO, an effective multiple curve comparison method. Our primary goal is to analyze protein folding trajectories, although the algorithm is general, and can be applied to compare multiple protein structures as well. Our new method greatly improved the performance of the POA algorithm by using a clustering preprocessing, a more discriminative two-level scoring function, as well as a novel merging post-processing procedure. It can detect low level of similarity among input curves. We demonstrated the effectiveness of our method by applying it to a set of simulated folding trajectories of the miniprotein Trp-cage. We also show the generality of our algorithm by using it to post-process the results returned by several current multiple protein structure alignment (MTSA) algorithms.

Currently, we have only experimented the EPO algorithm with a mini-protein (Trp-cage). One immediate question is to understand the scalability of the EPO algorithm for larger proteins or longer trajectories. In particular, a larger protein means a curve of higher dimensions. Our EPO algorithm seems to scale linearly with the number of participated trajectories, from current experiments. Furthermore, in practice, it is likely that we only perform the algorithm on small motifs. For longer trajectories, it seems that our algorithm scales in a quadratic manner. However, further experiments are necessary to investigate the scalability issue.

There are some previous work that analyze protein folding trajectories by collecting various statistics on measures such as the contact number (i.e, the number of native contacts) of each conformation along a trajectory and the URMS distance between a conformation and the native structure [13]. One way to view this is that a trajectory is mapped into a time-series data representing the evolution of, say, the number of native contacts, which can be considered as a one-dimensional curve. In this regard, we can use our EPO algorithm to analyze a collection of such curves induced by one measure. In general, there may be multiple measures, geometric or physio-chemical, that a user may wish to inspect. Hence it is highly desirable to build a framework for analyzing folding trajectories that can incorporate these multiple measures, and that also enables the addition of new properties easily. This is one important future direction for us.

Finally, at this stage, the EPO algorithm can only be used to find the best correspondences between (multiple) curves (protein structures) for fixed transformations. That is why current experiments in Section 5 only use the EPO to refine the MSTA results by other algorithms (so that there are good initial transformations). We are currently developing an independent MSTA software to align multiple protein structures based on the EPO algorithm (i.e, solving both the alignment and the superimposition problems). We also remark that compared to other multiple curve alignment algorithms, our algorithm is specifically effective at capturing low level of similarities. As such, another important future direction is to extract structural motifs from a family of proteins that may not share high global similarity.

## Methods

In this section, we describe our EPO algorithm for comparing a set of possibly high dimensional *general curves*. If we are given a set of protein folding data, we first convert each folding trajectory to a high dimensional curve. In particular, a folding trajectory is a sequence of conformations (structures) of a protein chain, representing different states of this protein at different time steps during the simulation of its folding process. We represent each conformation using the *distance map *between its alpha-carbon atoms so that it is invariant under rigid transformations. For example, if a protein contains *n *amino acids, then its distance map is a *n *× *n *matrix *M *where *M *[*i*] [*j*] equals the distance between the *i*th and *j*th alpha-carbon atoms along the protein backbone. This matrix can then be considered as a point in the *n*^2 ^dimensions. This way, we map each trajectory of *m *conformations to a curve in ${\mathbb{R}}^{n^{2}}$ with *m *vertices. We remark that one can also encode the side-chain information into the high dimensional curves, or map the trajectory of a substructure into a high dimensional curve. We will use such more refined high dimensional curves in most of our experiments as well. In the remaining part of this paper, we use the terms trajectories and curves interchangeably.

### Notations and algorithm overview

**Figure 5:**
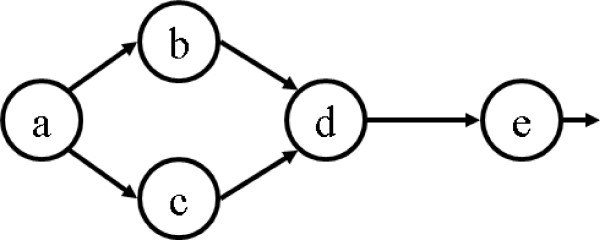
**A POG *G *of 5 nodes**. Note that there is a partial order constraint *a *≺*d *even though there is no edge between them. Both ⟨ *a*, *b*, *c*, *d*, *e*⟩ and ⟨*a*, *c*, *b*, *d*, *e*⟩ are valid partial order lists w.r.t. *G*.

Before we formally define the MCC problem, we introduce some necessary notations. Given a set of elements *V *= {*v*_1_,..., *v*_*l*_}, a relation ≺ over *V *is *transitive *if *v*_*i *_≺*v*_*j *_and *v*_*j *_≺*v*_*k *_imply that *v*_*i *_≺*v*_*k*_. In this paper, we also refer to *v*_*i *_≺*v*_*j *_as a *partial order constraint*. A *partial order graph *(POG) *G *= (*V*, *E*) is a directed acyclic graph with *V *= {*v*_1_,..., *v*_*l*_}, where *v*_*i *_≺*v*_*j *_if there is an edge (*v*_*i*_, *v*_*j*_). Note that by the transitivity of this relation, two nodes may have a partial order constraint even when there is no edge between them in *G*. Let *R *be the set of partial order constraints induced by *G*. We say that a permutation Π(*V*) of *V *is a *partial order list w.r.t. G *if for any *v*_*i *_≺*v*_*j *_∈ *R*, we have that *vi *appears before *v*_*j *_in the permutation Π(*V*). In other words, the linear order in Π(*V*) is a *total order *satisfying all partial order constraints induced from *G*. See Figure 5 for an example.

Let T = {*T*_1_,..., *T*_*N*_} be a set of *N *trajectories in ℝ^*d*^, where each trajectory *T*_*i *_is an ordered sequence of *n *points $p_{1}^{i},...,p_{n}^{i}$. (For simplicity, we assume without loss of generality that all *T*_*i*_s have the same length *n*.) The goal of the MCC algorithm is to find aligned sub-sequences from T.

**Figure 6:**
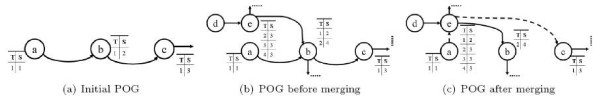
**POG construction**. Symbols inside the circles are the aligned node IDs. The table associated with each node encodes the set of points aligned to it. In particular, each row represents a point with its trajectory ID (**T **column) and its index along the trajectory (**S **column). For example, the entry (1, 2) associated with node *b *in (a) means that the aligned node *b *currently include the point $p_{2}^{1}$, the second point from trajectory-1. In (a), a POG is initialized by the trajectory *T*_1_. An example of a POG after aligning a few trajectories is shown in (b). Note that a new node/branch is created when a point cannot be aligned to any existing nodes. For example, node *e *was created when $p_{3}^{2}$ (i.e, the 3rd point of *T*_2_) was inserted. (c) shows the POG after merging point $p_{2}^{1}$ from the node *b *to the node *e *constrained by the distance threshold *ε*.

More formally, an *aligned node o *is a collection of vertices from *T*_*i*_s, with **at most **one point from each *T*_*i*_. Given a 3-tuple (T, *τ*, *ε*), where *τ *and *ε *are input thresholds, an alignment of T is a POG *G *with the corresponding set of partial order constraints *R *and a partial order list of aligned nodes O = {*o*_1_,..., *o*_*L*_} such that the following three criteria are satisfied:

C1. |*o*_*k*_| ≥ *τ*, for any *k *∈ [1, *L*];

C2. for any $p_{j}^{i},p_{j^{\prime }}^{i^{\prime }}\in o_{k}$, $||p_{j}^{i}-p_{j^{\prime }}^{i^{\prime }}||\leq \varepsilon$;

C3. if $p_{j}^{i}\in o_{k_{1}}$ and $p_{j^{\prime }}^{i^{\prime }}\in o_{k_{2}}$ with $o_{k_{1}}≺o_{k_{2}}$, then *j *<*j'*.

(C1) indicates that the number of vertices of input curves aligned to each aligned node *o*_*k *_is greater than a *size threshold τ *. (C2) means that these aligned points are tightly clustered together (i.e, the diameter of them is bounded by a *distance threshold ε*). (C3) enforces that points in different aligned nodes still maintain their partial order along their respective trajectory, which means that *o*_*k*_s are inherited and thus consistent to the points in each trajectory. Our goal is to maximize *L*, the size of such an alignment O. See Figure 6(b) for an example of an alignment graph.

#### Algorithm overview

At a high level, the EPO algorithm has two stages (see Figure 6): (S1) initial POG construction stage and (S2) merging stage. The first stage generates an initial alignment for T, encoded in a POG *G*. The procedure has the same framework as the POA algorithm, but its performance, especially when the similarity is low, is significantly improved, via the use of a clustering preprocessing step and a new two-level scoring function. In the second stage, we develop a novel and effective procedure to merge nodes from *G *to output a better final alignment G*. Below, we describe each stage in detail.

### Initial POG construction

Standard dynamic programming (DP) [35,36] is an effective method for pairwise comparison between sequences. It produces an optimal alignment between two sequences with respect to a given scoring function. One can perform multiple sequences alignment progressively based on this DP pairwise comparison method. Roughly speaking, in the *i*th round of the algorithm, the alignment of the first *i *– 1 sequences is represented in a consensus sequence. The algorithm then updates this consensus by aligning it with the *i*th sequence *S*_*i *_using the standard DP algorithm. Information from *S*_*i *_that is not aligned to the consensus sequence isessentially lost. See Figure 1(a).

The partial order alignment (POA) algorithm [3] alleviates this problem by encoding the consensus in a POG instead of a linear sequence (see Figure 1(b)). That is, the alignment of *S*_1_,..., *S*_*i*-1 _is encoded in a partial order graph *G*_*i*_, which is then updated to *G*_*i*+1 _by aligning it with *S*_*i*_. Due to the partial order in a POG, the alignment between *G*_*i *_and *S*_*i *_can still be achieved by a DP algorithm. The POA algorithm reduces the influence of the order of the sequences aligned, and is able to capture alignments between a subset of sequences. More details of the POA algorithm and its variants can be found in [2,3].

In our case, each trajectory is mapped to an ordered sequence of points (i.e, a polygonal curve), and a similar algorithm can be applied to our trajectory data: Instead of the usual 1D sequences, we now have *dD *sequences, where *d *is the dimension of each point. Note that since each point corresponds to the distance map of a conformation, no transformation is needed when comparing such curves. The first stage of our EPO algorithm constructs a POG *G *with respect to the input set of trajectories T using a modified POA algorithm. Below we explain the main differences between them.

#### A clustering preprocessing stage

The first problem with the standard POA algorithm is that the size of the POG graph maintained expands quickly when the level of similarity is low. For example, suppose we are updating the current POG *G*_*i *_to *G*_*i*+1 _by aligning it with a new curve *T*_*i*_. If a point *p *∈ *T*_*i *_cannot be aligned to any node in *G*_*i*_, then it will create a new node in *G*_*i*+1_, as this node may potentially be aligned later with the remaining curves. Consequently, if the similarity is sparse, many new nodes are created without really producing densely aligned nodes later and the size of the POG graph increases rapidly. This induces high computational complexity.

To address this problem, our algorithm first preprocesses all points from the input curves T by clustering them into groups [37], the diameter of which is smaller than a user defined threshold, which is fixed as the distance threshold *ε *in our experiments. According to the clustering result, we only keep those points that belong to a cluster holding more than *τ *curves' points in it. For example, if the threshold is *τ *= 3 (i.e, we require each aligned node aligns points from at least 3 curves), we will prune out the points in such clusters that cover less than 3 curves. Meanwhile we collect cluster centers in *C *= {*c*_1_,..., *c*_*r*_}, which we refer to as the *set of canonical cluster centers*. Intuitively, *C *provides a synopsis of the input curves and represents potentially aligned nodes.

If, in the process of aligning *T*_*i *_with *G*_*i*_, a point *p *∈ *T*_*i *_is not aligned to any node in *G*_*i*_, then we insert a new node in *G*_*i*+1 _**only if ***p *is within *ε *away from some canonical center from *C *– if *p *is far from all the canonical cluster centers, then there is little chance that *p *can form significant alignment with points from later curves, as that would have implied that *p *should belong to a dense cluster. We remark that this *set of canonical cluster centers *are not only used for shrinking the size of POG, but also used as a predictor of the new two-stage scoring function that we will introduce shortly. There is also further advantage of pruning out unpromising points in the second merging stage of the EPO algorithm.

#### Scoring function

The choice of the scoring function when aligning *G*_*i *_= (*V*_*i*_, *E*_*i*_) with *T*_*i*_, is in general a crucial aspect of an alignment algorithm. A good scoring function will align as many points as possible globally. Given a point *p *∈ *T*_*i *_and a node *o *∈ *G*_*i*_, let *δ*(*o*, *p*) be the similarity between *p *and *o*, the definition of which will be described shortly. The *score *of aligning *p *with *o *is usually defined as:

$$\begin{array}{l} \text{Score}(\text{o},\text{p})=\\ \begin{array}{ll} \max & \{\underset{(o^{\prime },o)\in E_{i}}{\max}(\text{Score}(\text{ o}\prime ,\text{q})+\delta (\text{o},\text{p})),\\ & \begin{array}{cc} \underset{(o^{\prime },o)\in E_{i}}{\max}\text{Score}(\text{ o}\prime ,\text{p}), & \text{Score}(\text{o},\text{q})\}, \end{array} \end{array} \end{array}$$

where *q *is the parent of the point *p *along *T*_*i*_, and *o' *ranges over all immediate predecessors of *o *in the POG *G*_*i*_. It is easy to verify that such scores can be computed by a dynamic programming procedure due to the inherent order existing in both the trajectory and the POG.

A common way to define *δ*(*o*, *p*), the similarity between *o *and *p*, is as follows. Assume that each node *o *is associated with a *node center ω *(*o*) to represent all the points aligned to this node. Then

(1) $$\delta (o,p)=\{\begin{array}{ll} \varepsilon -||p-\omega (o)|| & \text{if}||p-\omega (o)||<\varepsilon \\ 0 & \text{otherwise} \end{array}$$

An alternative way to view this is that each node *o *has an influence region of radius *ε *around its center. A point *p *can be aligned to a node *o *only if it lies within the influence region of *o*.

**Figure 7:**
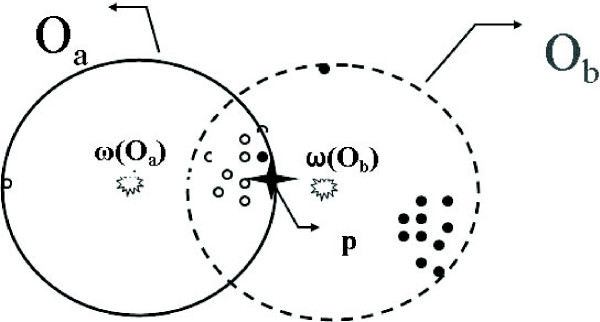
**An example for scoring function**. Empty and solid points are aligned to the nodes *o*_*a *_and *o*_*b*_, respectively. For a new point *p *(the star), although it is closer to *ω*(*o*_*b*_), it is better grouped with points aligned to *o*_*a*_. Hence ideally, it should be aligned to *o*_*a *_instead of to *o*_*b*_.

Natural choices for the node center *ω*(*o*) of *o *include using an earlier computed canonical cluster center, or the center of the minimum enclosing ball of points already aligned to this node (or some weighted variants of it), which is a dynamic point relying on alignment order. The advantage of the former is that canonical cluster centers tend to spread apart, which helps to increase coverage of aligned nodes. Furthermore, the canonical cluster centers serve as good candidates for node centers as we already know that there are many points around them. The disadvantage is that it does not consider the distribution of points already aligned to this node. See Figure 7, where without considering the distribution of points aligned to *o*_*a *_and *o*_*b*_, the new point *p *will be aligned to *o*_*b *_even though *o*_*a *_is a better choice. Using the center of the minimum enclosing ball alleviates this problem. However, the influence regions of nodes produced this way tend to overlap much more than using the canonical cluster centers and the position of these centers also depend heavily on the order of curves aligned. We combine the advantages of both approaches into the following two-level scoring function for measuring the similarity *δ*(*o*, *p*).

Specifically, for a node *o*, let *q *be the first point aligned to this node. This means that at the time we were examining *q*, *q *cannot be aligned to any existing node in the POG. Let *c*_*k *_∈ *C *be the nearest canonical cluster center of *q *– recall that the node *o *was created because ||*q *– *c*_*k*_|| ≤ *ε*. We add *c*_*k *_as a *point *aligned to this node, and at any time, the center of the minimum enclosing ball of currently aligned points, *including c*_*k*_, will be used as the node center *ω*(*o*). Now let

$$D(o)=\underset{q,q^{\prime }\in o}{\max}||q-q^{\prime }||$$

be the diameter of points currently aligned to *o*. We define that:

(2) $$\delta (o,p)=\{\begin{array}{ll} 2\varepsilon & \text{if}||p-\omega (o)||<D(o)\\ \varepsilon & \text{else if}||p-\omega (o)||<\varepsilon \\ 0 & \text{else} \end{array}$$

In other words, the new scoring function prefers centering points to be around previously computed cluster centers, thus tending to reduce overlaps between the influence regions of different nodes. Furthermore, it gives higher similarity score for points that are more tightly grouped together with those already aligned at current node, addressing the problem shown in Figure 7. Our experimental tests have shown that this two level scoring function significantly outperforms the ones using either only the canonical centers or only the centers of minimal enclosing balls. We remark that it is possible to use variants of the above two-level scoring function, such as making it continuous (instead of being a step function). We choose the current form for its simplicity. Furthermore, experiments show that there is only marginal difference if we use the continuous version.

### Merging stage

In the first stage, we have applied a progressive method to align each trajectory onto an alignment graph one by one. In the *i*th iteration, a point from *T*_*i *_is either aligned to the best matched node in the current POG *G*_*i*_, or a new node is created containing this point and the corresponding canonical cluster center, or discarded. After processing all of the *N *trajectories in order, we return a POG *G *= *G*_*N *_. In the second stage of our EPO algorithm, we further improve the quality of the alignment in *G *by using a novel merging process.

Given the greedy nature of the POA algorithm, the alignment obtained in *G *is not optimal and depends on the alignment order. Furthermore, since the influence regions of different aligned-nodes may overlap, no matter how we improve the scoring function, sometimes it is simply ambiguous to decide locally where to align a new in-coming point, and a wrong decision may have grave consequence later.

**Figure 8:**
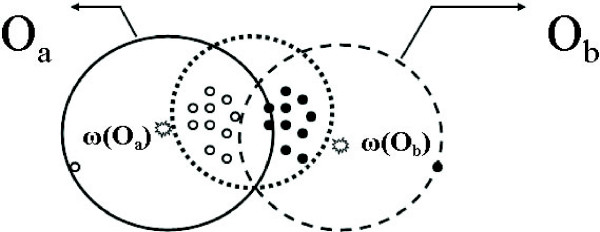
**An example for merging stage**. Empty and solid points are aligned to the nodes *o*_*a *_and *o*_*b*_, respectively, while points in the dotted region should be grouped together.

For example, see Figure 8, where the set of points *P *(enclosed in the dotted circle) should have been aligned to one node. However, suppose the nodes *o*_*a *_and *o*_*b *_already exist before any point in *P *is inserted.

Then as points from *P *come in, it is rather likely that they are distributed evenly into both *o*_*a *_and *o*_*b*_. This problem becomes much more severe in higher dimensions, where *P *can be distributed to several nodes whose centers are well-separated around *P*, but whose influence regions still cover some points from *P *(the number of such regions grows exponentially w.r.t. the dimension *d*). Hence instead of being captured in one heavily aligned node, *P *is broken into many nodes with small size. Our experimental tests confirm that this is happening rather commonly in both standard and our modified POA algorithms.

To address this problem, we propose a novel postprocessing on *G*. The goal is to merge qualified points from neighboring less-aligned nodes to augment more heavily loaded nodes. In particular, the following two invariants are maintained during the merging process:

(I1) At any time, the diameter of the target node is still bounded by the distance threshold *ε*;

(I2) The partial order constraints induced by the POG graph are always consistent with the order of points along each trajectory.

The second criterion means that at any time in the POG graph *G'*, if *p *∈ *o*_1_, *q *∈ *o*_2_, *p*, *q *∈ *T*_*i *_and *p *precedes *q *along the trajectory *T*_*i*_, then either *o*_1 _≺*o*_2_, or there is no partial order relation between them. In other words, the resulting POG still corresponds to a valid alignment of T with respect to the same thresholds.

As an example, see Figure 6, where the point $p_{2}^{1}$ (i.e, the second point of the trajectory *T*_1_) in the node *b *in (b) is moved to the node *e *in (c). Note that the graph is also updated to reflect the change (the dashed edge in (c)), in order to maintain the invariants (I1) and (I2). When all points aligned to a specific node *o *are merged (thus moved) to other nodes (i.e, *o *becomes empty), we delete *o*, and its successors in the POG will then become the successors of its parent.

**Figure 9:**
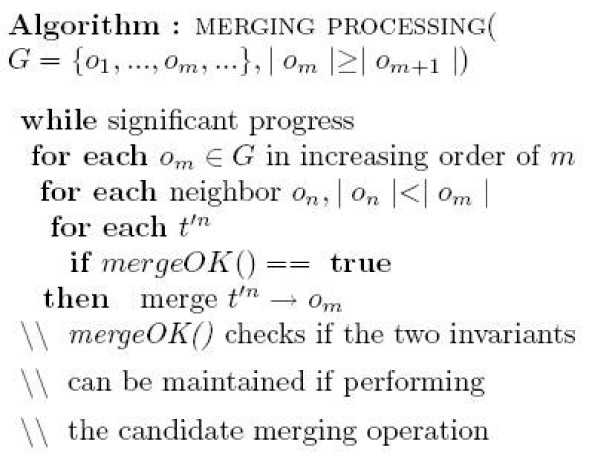
The merging algorithm.

A high level pseudocode of the merging process is shown in Figure 9. It augments better aligned nodes from the current POG *G *by processing first the nodes with larger size. We perform this procedure a few times till there is no significant increase in the quality of the resulting alignment. In practice, to speed up the algorithm, we merge neighbors to a node *o *only if its size is greater than some threshold (fixed at half of the size threshold, i.e, *τ*/2, in our experiments), as otherwise, there is low probability that *o *will become a heavy node later.

## Authors' contributions

HS and YW carried out the algorithm development, as well as drafted the manuscript. HS also implemented the algorithm and carried out all experiments. HF and YW conceived of the study, provided critical input in design and coordinated various aspects of the project. MO provided the experimental data. All authors read and approved the final manuscript.
